# The Influence of Preoperative Oral Carbohydrate Administration on the Perioperative Period in Children and Adolescents After Orthopedic Procedures—A Pilot Study

**DOI:** 10.3390/healthcare14030361

**Published:** 2026-01-30

**Authors:** Iwona Zaporowska-Bugajewska, Tomasz Mazurek, Szymon Wałejko, Justyna Napora, Wioletta Mędrzycka-Dąbrowska

**Affiliations:** 1 Clinical Department of Orthopaedics and Traumatology of the Musculoskeletal System, COPERNICUS Medical Entity, 80-803 Gdańsk, Poland; jzaporowska@wss.gda.pl (I.Z.-B.); mazurek@gumed.edu.pl (T.M.); szymon.walejko@gumed.edu.pl (S.W.); justanapora@gmail.com (J.N.); 2 Department of Orthopaedics and Traumatology, Faculty of Medicine, Medical University of Gdansk, 80-210 Gdańsk, Poland; 3 Department of Anaesthesiology Nursing & Intensive Care, Faculty of Health Sciences, Medical University of Gdansk, Debinki 7, 80-211 Gdańsk, Poland

**Keywords:** perioperative care, pediatric orthopedics, oral polycarbohydrate preparations

## Abstract

Background: Preoperative fasting is one of the most difficult stages of hospitalization for children and their caregivers. The popularization of preoperative oral polycarbohydrate preparations is an important element influencing the comfort of hospitalized children. The aim of the study was to assess the effect of carbohydrate administration on perioperative blood glucose (PBG), the occurrence of complications in the pre- and postoperative periods, and satisfaction in children and adolescents. Material and methods: The study was a comparative-observational one, and the following research methods were used: an author’s diagnostic questionnaire, observation, and analysis of medical documentation. The research group consisted of 50 patients from the Pediatric Orthopedic Department who received an oral polycarbohydrate solution up to 2 h before anesthesia. The control group consisted of 50 patients who fasted for more than 6 h before anesthesia. The study was conducted between February and May 2024. Results: The patients were assessed using a measurement of venous blood glucose taken immediately before the administration of premedication. Parents completed a questionnaire regarding their child’s perioperative period. Conclusions: The glucose level in patients who received a polycarbohydrate preparation is higher than in children who did not. The administration of polycarbohydrate preparations influences the feeling of thirst and hunger in the postoperative period in children and adolescents. Oral administration of a polycarbohydrate preparation up to 2 h before anesthesia does not cause regurgitation in children and adolescents. Patients who received an oral polycarbohydrate preparation tolerated the waiting period before anesthesia better. Preoperative fasting is, apart from the insertion of venous access, the most stressful situation for children and adolescents during hospitalization. The supply of oral polycarbohydrate preparations has a positive effect on pediatric patients in the perioperative period.

## 1. Introduction

Hospitalization and preparation of a child for surgery is an extremely stressful period for both the child and the caregiver. Preoperative education includes information on fluid and food intake and required fasting before anesthesia. In clinical practice, it is evident how difficult it is for children to follow the recommendations related to fasting. Surveillance of pediatric patients while awaiting anesthesia must be carefully planned and strictly implemented. Parents accompanying the child during hospitalization become members of the team responsible for preparing the patient for safe anesthesia in conditions that are as comfortable as possible for the child. A key factor influencing effective cooperation in this area is the child’s age and intellectual level, which is not always consistent with biological age [1,2].

Traditionally, preoperative fasting has been the standard practice for patients undergoing elective surgery, primarily aimed at reducing the risk of pulmonary aspiration [3,4]. Preoperative oral carbohydrate consumption can improve postoperative recovery in patients fasting for various types of surgery [5]. Literature shows that midnight fasting does not reduce the volume or acidity of gastric contents associated with perioperative complications [5,6]. Surgical interventions trigger a cascade of catabolic reactions characterized by the release of stress hormones such as cortisol and the activation of proinflammatory pathways. In contrast, preoperative fasting increases metabolic stress, which can lead to insulin resistance and postoperative symptoms such as thirst, hunger and anxiety, all of which are associated with prolonged hospital stays [5,6,7]. Preoperative oral polycarbohydrate administration (POC) has been shown to improve patient well-being and accelerate postoperative recovery. The Enhanced Postoperative Recovery Scheme (ERAS) is a strategic approach designed to mitigate metabolic disturbances and improve patient comfort during the perioperative period. ERAS protocols recommend preoperative oral polycarbohydrate (POC) intake to alleviate surgical stress and counteract the adverse effects of fasting [8,9,10]. However, evidence regarding its effectiveness in orthopedic patients remains insufficient and requires further research [7].

### Aim

The aim of the study was to assess the effect of polycarbohydrate administration on perioperative blood glucose (PBG), the occurrence of complications in the pre- and postoperative period, and satisfaction in children and adolescents.

## 2. Materials and Methods

### 2.1. Study Design

A comparative observational study was carried out between February and May 2024 after obtaining the approval of the Bioethics Committee of the Medical University of Gdansk (decision KB/744/2023–2024). The guidelines of the Helsinki Declaration (World Medical Association, 2014), STROBE (Strengthening the Reporting of Observational Studies in Epidemiology) [11], and the General Data Protection Regulation [12] were followed.

### 2.2. Participants

**Figure 1:**
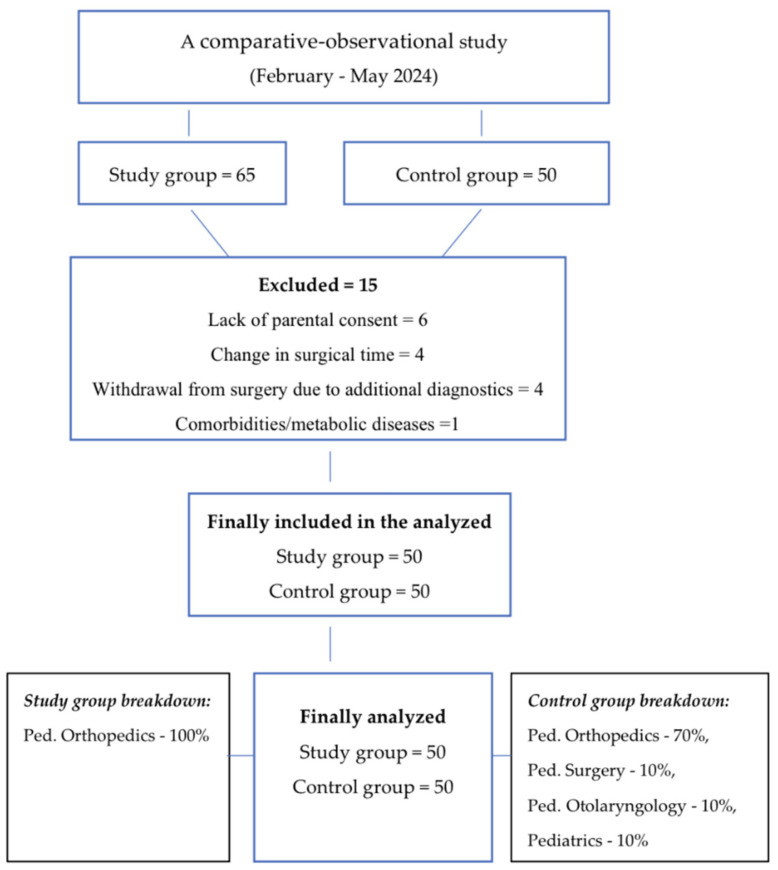
Diagram Flow.

The study site was the Pediatric Orthopaedics Department, where patients qualified for the study group and some patients from the control group were analyzed. In addition to the Department of Orthopaedics, due to the lack of standard use of polycarbohydrate oral fluid for patients, the control group was recruited in Departments of Pediatrics, the Department of Otolaryngology, and the Department of Pediatric Surgery (Figure 1).

The study included a group of 50 randomly selected patients aged 3 to 18 years, with normal body weight for their age and sex, without coexisting metabolic diseases, who received an oral polycarbohydrate preparation up to 2 h before anesthesia, in an amount ranging from 100 to 200 mL depending on mass, according to producer recommendations. Due to the pilot nature of the study, no placebo was administered to the control group. All patients were scheduled for planned inpatient surgical procedures. Preparation for anesthesia required a 6 h fasting period before surgery, as per anesthesiological instructions. All study participants received general anesthesia with airway protection (endotracheal tube). No antiemetic prophylaxis was used.

### 2.3. Inclusion and Exclusion Criteria

Inclusion criteria:

✓ Obtaining written consent from the patient’s parent, 6 h fasting period before anesthesia and oral intake of the polycarbohydrate preparation up to 2 h before anesthesia—study group.
✓ Obtaining written consent from the patient’s parent and a 6 h fasting period before anesthesia—control group.

Exclusion criteria:

✓ Lack of parental consent.
✓ Change in surgical time.
✓ Withdrawal from surgery due to additional diagnostics.
✓ Comorbidities/metabolic diseases.

### 2.4. Research Tools

The research techniques used in the study included the analysis of diagnostic tests assessing blood glucose levels, an observational protocol, and a questionnaire survey.

Due to the lack of standardized tools that would enable the acquisition of information relevant to the research procedure, the following were constructed:

▪ A custom-designed questionnaire: consisting of a demographic section containing the following data: age, gender, body weight, and height; and a main section: closed-ended questions regarding the patient’s subjective experiences, the perioperative period, and feelings of hunger and thirst.
▪ An observational protocol containing information regarding venous blood glucose levels before and after premedication administration, reported feelings of hunger and thirst, the occurrence of regurgitation and nausea, and patient behaviour.

An additional tool used in the research procedure was a questionnaire developed specifically for the study, addressed to the patient’s caregiver/parent, regarding the assessment of the perioperative period.

### 2.5. Data Collection and Research Sample

To ensure the reliability of the results, the sample size was determined based on the significance level (α—0.05) and standard deviation (SD). In 2024, 850 children underwent surgery at the Children’s Orthopedic Department. The research group consisted of 50 children, which is 6% of the population and meets the requirements necessary to detect an effect in studies comparing two groups. Based on the size of the historical group, the size of the control group was determined—50 patients. Therefore, the size of the groups is justified on a substantive basis.

**Figure 2:**
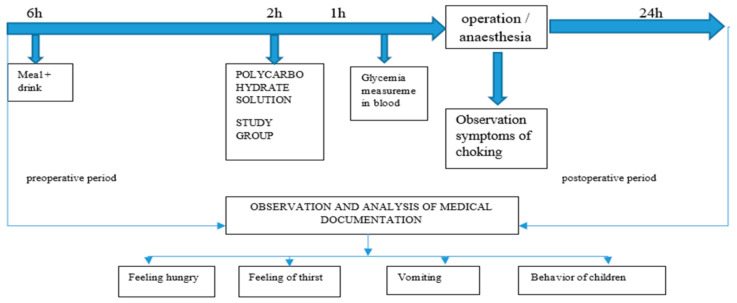
Research process scheme.

The research process for patients in both groups, the experimental and the control, was strictly defined and included post-operative care. The only differentiating factor was the oral administration of the polycarbohydrate preparation (Figure 2).

### 2.6. Statistical Analysis

Calculations for the purpose of this study were performed using Statistica 13.1 PL, and the charts were prepared in Microsoft Excel^©^. Frequency statistics, including number (n) and percentage (%), were used in the analysis. Due to the qualitative nature of most variables, relationships between these characteristics were verified using Pearson’s chi-squared test of independence. For variables measured on an ordinal scale, the Mann–Whitney U test was applied. In two cases involving dependent variables measured on a quantitative scale, the applicability of parametric testing was assessed. When the dependent variable demonstrated a normal distribution (verified by the Shapiro–Wilk W test) and homogeneity of variance (Levene’s test), the parametric Student’s *t*-test was used. When these assumptions were not met, the Mann–Whitney U test was applied. The significance level was set at α < 0.05.

## 3. Results

### 3.1. Socio-Demographic Factors

The research material consisted of 100 observations. The subjects were divided into two equal groups. The first group, the control group, consisted of patients up to 18 years of age who did not receive polycarbohydrate preparations during preoperative preparation. The study group included children and adolescents who were given oral polycarbohydrate preparations.

In terms of age and gender, the two groups were similar. In both groups, boys were slightly predominant, accounting for 52% of the control group and 58% of the study group. Girls constituted 48% and 42% of the respective groups. Regarding age, the study group included averaged 14.18 years, while the control group had a mean age of 12.36 years. There were no statistically significant differences between the groups in terms of age or gender.

### 3.2. Glucose Level

Medical data collected during the study included the children’s venous blood glucose levels before anesthesia. The comparison of the groups in this respect (Table 1) yielded a statistically significant result. The children’s blood glucose levels were strongly (d = 1.11) associated with the intake of polycarbohydrate. Significantly higher glucose levels were observed in children who received an oral multi-sugar product.

### 3.3. Feelings of Hunger and Thirst

**Figure 3:**
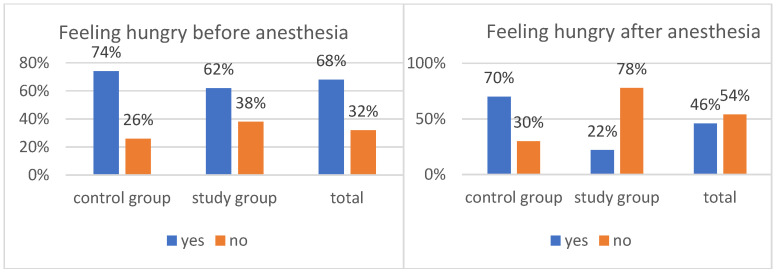
Reported feelings of hunger before and after anesthesia.

**Figure 4:**
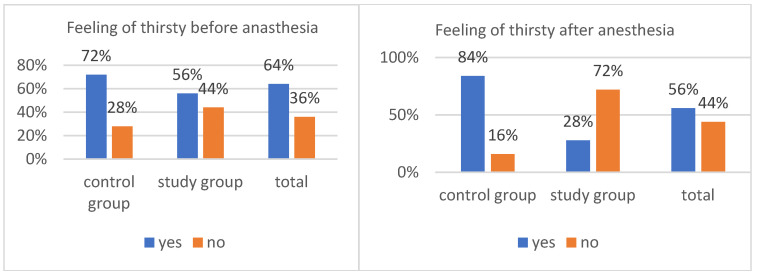
Feeling of thirst before and after anesthesia.

The study also collected information on feelings of hunger and thirst in children before and after anesthesia. Overall, 46% of the children reported feeling hungry, and 56% of respondents reported feeling thirsty (Figure 3 and Figure 4).

The information obtained from the patient observation cards, which expanded upon earlier findings, formed the basis for verifying several detailed hypotheses. Most pediatric patients awaiting general anesthesia reported feeling hungry, a sensation experienced by 64% of the respondents.

Data presented in Figure 3 indicate that most children in the period before anesthesia complained to their parents or nurse about feeling hungry. The overall percentage of patients reporting hunger was 68%. Administration of polycarbohydrate formulations was irrelevant here, chi^2^(1) = 1.65, *p* = 0.198. In both groups, the percentages of children complaining of hunger were similar: 74% in the control sample and 62% in the study sample.

The feeling of hunger differed between children in the control group and those in the study group who received oral polycarbohydrate preparations, χ^2^(1) = 23.19, *p* < 0.001. The strength of the relationship between the variables was moderate, φ = 0.48. Children in the control group were significantly more likely to report hunger after anesthesia (70%) compared with children in the study group (22%). When comparing the data within individual groups, the result approached statistical significance, χ^2^(1) = 2.78, *p* = 0.096. Preoperative hunger was more commonly reported among children who did not receive polycarbohydrate preparations (72%) than among those who did (56%). In total, 64% of patients reported feeling hungry to their parents or nurses before anesthesia.

The feeling of thirst immediately before anesthesia was observed in 63% of cases (Figure 4). It occurred in 76% of the children in the control group and in 50% of the children in the study group. This difference was statistically significant, χ^2^(1) = 7.25, *p* = 0.007. The level of thirst was significantly higher in children who were not given oral polycarbohydrate preparations than in those who received them. The relationship between the analyzed variables was of low strength, φ = 0.27.

In the case of thirst in the postoperative period (Figure 4), the intake of oral polycarbohydrate preparations was significant, χ^2^(1) = 41.03, *p* < 0.001. The strength of the relationship was high (φ = 0.64), and thirst was much more common in children who were not given a polycarbohydrate preparation (84%) than in those who received it (20%). The overall percentage of patients reporting thirst in the postoperative period was 52%.

The administration of polycarbohydrate made an even greater difference in the sensation of thirst after anesthesia, χ^2^(1) = 31.82, *p* < 0.001. The association between these variables was strong, φ = 0.56. Children who were not given the above-mentioned preparations were significantly more likely to report thirst after anesthesia (84%) compared with children who received them (28%).

### 3.4. Regurgitation

**Figure 5:**
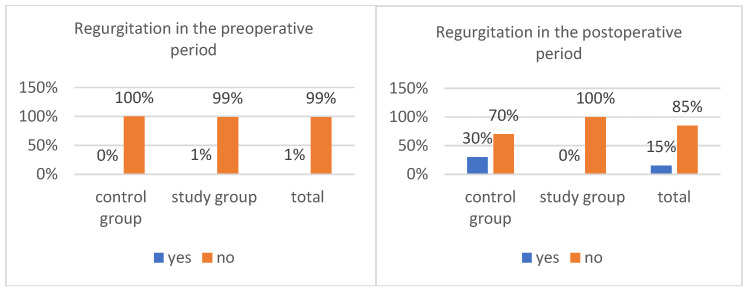
Regurgitation in the pre and postoperative period.

In the period before anesthesia and surgery, only one child (1%) experienced regurgitation. This case occurred in a child receiving an oral polycarbohydrate preparation, representing 2% of the study group (Figure 5). Regurgitation occurred more than one hour before administration of the product and did not occur after administration of the fluid; therefore, it did not constitute grounds for excluding the patient from the study. No cases of regurgitation were reported in the control group. The difference between the groups was not statistically significant, χ^2^(1) = 0.01, *p* = 0.315. Administration of the preparation was not associated with the frequency of regurgitation among children undergoing surgery.

After anesthesia, some children (15%) reported regurgitation (Figure 5). It was found that regurgitation occurred only among children who had not been given a polycarbohydrate preparation (30%). No regurgitation was observed in the study group (0%). Analysis of the results showed that this difference was statistically significant, χ^2^(1) = 17.65, *p* < 0.001. Patients who received an oral preparation containing multisugars vomited significantly less frequently in the post-anesthesia period than those who did not receive such a product. The strength of the relationship between the variables was moderate, φ = 0.42.

### 3.5. Symptoms of Choking

**Figure 6:**
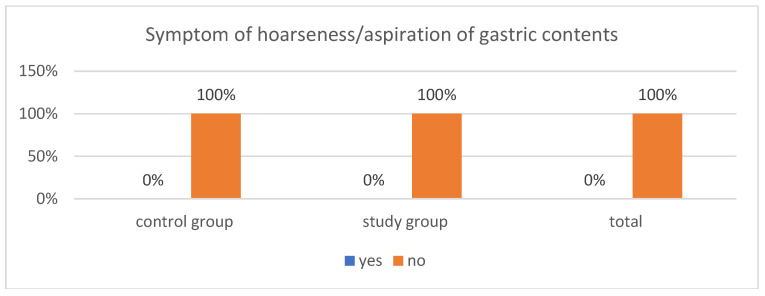
Symptom of hoarseness/aspiration of gastric contents.

Data on symptoms of hoarseness or aspiration of gastric contents during anesthesia are presented in Figure 6. None of the patients in either group exhibited symptoms of intraoperative hoarseness. Safety in this regard is a fundamental element of pre-anesthesia preparation. Strict adherence to the amount and timing of product administration, along with careful documentation of this information, is one of the essential responsibilities of the nurse caring for the patient.

### 3.6. Reactions of Children and Adolescents to the Supply of Polycarbohydrate Preparations

**Figure 7:**
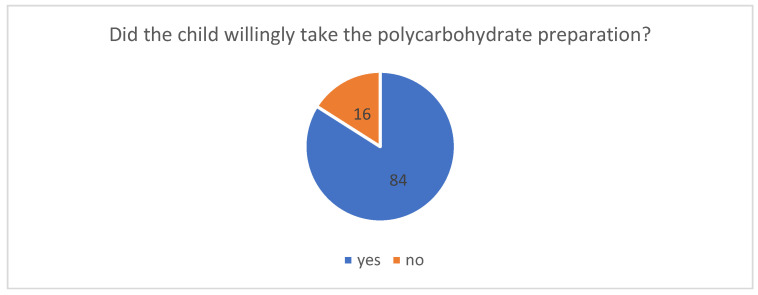
Did the child willingly take the polycarbohydrate preparation?

The survey conducted for the purposes of the study included a question regarding the reactions of children and adolescents to the administration of polycarbohydrate preparations. The results obtained in this respect are presented below. The data shown in the graph (Figure 7) indicate that the clear majority of children (84%) willingly accepted the oral polycarbohydrate preparation before the procedure. Sixteen percent of patients responded reluctantly, which in all cases was due to poor tolerance of the product’s taste.

### 3.7. The Behaviour of Children

**Figure 8:**
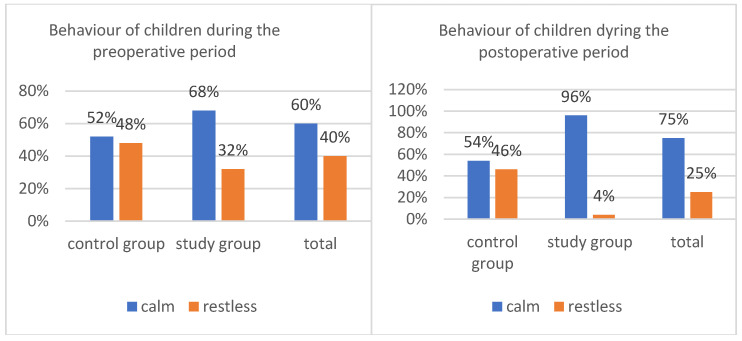
Behaviour of children during the pre and postoperative period.

The behaviour of the majority of children (60% of the total) in the preoperative period was non-objectionable, and the patients remained calm (Figure 8). The administration of the polycarbohydrate formulation was not a significant factor, χ^2^(1) = 2.67, *p* = 0.102. The percentage of calm patients awaiting anesthesia was 52% in the control group and 68% in the study group.

The children are characterized by greater calmness after awakening from anesthesia, *chi*^2^(1) = 23.52, *p* < 0.001, *fi* = 0.48. Children who received the product were significantly more likely (96%) to behave calmly and were willing to cooperate or sleep than children who did not receive the product (54%). Overall, three out of four (75%) patients were calm after surgery (Figure 8).

## 4. Discussion

In the United States, as early as 1941, Pearson described “serious emotional reactions in children undergoing anesthesia and surgery” [13], while interest in perioperative stress in children emerged in Europe many years later. Observations by Litke et al. indicate that in many centres in Poland, knowledge about supporting children and their parents is insufficient, and the approach to treatment remains overly objective [1].

### 4.1. Glucose Level

The difference between venous blood glucose levels in children in the study and control groups was statistically significant. Significantly higher glucose levels were found in children who received an oral multi-sugar product. Fasting prior to surgery reduces the body’s glycogen stores, induces a negative fluid balance, and increases catabolic processes before the procedure [14]. In the subsequent stage of the neurohormonal response, insulin resistance develops, which is the most important adverse manifestation of the metabolic response to injury. Hyperglycemia resulting from this process increases the risk of complications and prolongs the duration of hospitalization [15,16]. For this reason, any measures that minimize these adverse reactions are of great importance. Fluctuations in blood glucose levels during the postoperative period negatively affect patient behaviour, feelings of intensified hunger, and the healing process of the postoperative wound [17]. In the present study, a single measurement of venous blood glucose was performed immediately before premedication; however, extending the observation to include postoperative glucose measurements and correlating them with patient behaviour may be considered.

### 4.2. Feeling Hungry and Thirst

The frequency of children’s feelings of hunger and thirst in the perioperative period is associated with the oral administration of polycarbohydrate preparations. It has been shown that oral administration of a 12.5% solution of maltodextrin to children and adolescents prior to general anesthesia has a positive effect on patient behaviour and comfort—reducing feelings of hunger and thirst, decreasing the frequency of regurgitation during the perioperative period, and being positively perceived by parents. Similar conclusions were reported by Ying et al., who analyzed the behaviour of 303 children. A group of 152 patients received a preparation containing multisugars during the preoperative period. Compared with patients who did not receive oral polycarbohydrate supplementation, children in the study group reported less thirst and were more satisfied with their care [2,18]. It should be emphasized that the association between the administration of these preparations and reduced feelings of hunger and thirst was demonstrated in both the preoperative and postoperative periods.

### 4.3. Regurgitation

In terms of the frequency of regurgitation during the perioperative period, it was observed that patients who were given an oral preparation containing polycarbohydrate vomited significantly less frequently during the post-anesthesia period than children who were not given this type of product. The strength of the relationship between the variables was moderate (φ = 0.42). Postoperative nausea and regurgitation (PONV) is one of the most common complications after general anesthesia and has a significant impact on postoperative comfort. Postoperative regurgitation can lead to complications such as aspiration of gastric contents. Frykholm et al. found that when children are given oral fluids early, the incidence of postoperative regurgitation increases [19]. Therefore, the absence of thirst resulting from preoperative administration of polycarbohydrate is a beneficial factor, as it supports delaying the postoperative administration of oral fluids.

### 4.4. Symptoms of Choking

The risk of regurgitation or aspiration of gastric contents into the bronchial tree is one of the most frequently analyzed elements of ERAS procedures [3,18]. None of the patients in either group exhibited symptoms of intraoperative hoarseness. Ensuring safety in this regard is a fundamental component of preparing patients for anesthesia. In the available literature, no reports have indicated an increased risk of aspiration into the bronchial tree among patients who received an oral polycarbohydrate solution 2 h before the procedure. A review of studies involving 140,000 sedated children and adolescents conducted by van der Putta et al. showed that the frequency of aspiration was similar in patients fasting for more than 6 h and those receiving oral fluids up to 2 h before anesthesia. The oral administration of clear fluids is therefore not a factor that increases the risk of regurgitation or aspiration into the bronchial tree [11,20].

Recent reports published in the *British Journal of Anesthesia* indicate that giving clear fluids even 1 h before anesthesia in children and adolescents does not increase the risk of aspiration into the airways [14]. Studies evaluating gastric emptying using ultrasound confirm that a 1 h interval is safe after oral administration of clear fluids in amounts adapted to the child’s body weight—10–15 mL/kg. According to Beck et al., oral polycarbohydrates can be used in different age groups, taking the child’s body weight into account [21,22]. Schmitz et al. published the results of a multicenter prospective study on regurgitation and aspiration during sedation. The findings from Schmitz’s study also indicate that there is no evidence of an association between these complications and the intake of an oral polycarbohydrate solution, which is further supported by research presented by Gianotti [23,24,25].

### 4.5. Reactions of Children and Adolescents to the Supply of Polycarbohydrate Preparations

The results show that the children drank the polycarbohydrate solution without hesitation. The vast majority of children (84%) were willing to take the oral preparation before the procedure. The ability to drink a liquid and the shortening of the preoperative fasting period elicited positive reactions. Sixteen percent of patients responded reluctantly, which in all cases was due to poor tolerance of the product’s sweet taste. Studies have also described positive reactions in children to the preoperative oral administration of a beverage containing polycarbohydrate [26]. There are, however, few studies on the preoperative oral administration of polycarbohydrate to children, and these are often preliminary reports.

### 4.6. The Behaviour of Children

Improving patient comfort during the perioperative period and ensuring parental satisfaction, which translates into better cooperation and active participation in the therapeutic process, is one of the main priorities of modern inpatient hospitalization. One of the key aspects analyzed in the present study is the reduction in stress experienced by children and their parents during hospitalization. Care that minimizes the emotional stress of both the patient and the caregiver should constitute a general standard of practice for medical personnel [1,2].

The analysis focused on the patient’s well-being and behaviour, as well as the satisfaction of the accompanying parent during hospitalization. Calm behaviour—defined as the absence of psychomotor agitation in the child during the preoperative period—depends on multiple factors [6]. Based on the analysis of information obtained from parents in both the study and control groups, it can be concluded that the requirement to fast is one of the most frequently mentioned inconveniences experienced by children during hospitalization. Similar findings are widely reported in the literature. Numerous studies have demonstrated that preoperative fasting is associated with irritability, frequent crying, and parental anxiety in the hospital setting [3,27,28]. Cheng et al. conducted a meta-analysis of randomized trials in 2021 that included a total of 5606 patients. The analysis addressed the impact of preoperative administration of oral polycarbohydrate solutions on patient well-being, including dry mouth, feelings of hunger and thirst, regurgitation, symptoms of regurgitation/aspiration, and overall satisfaction with hospitalization [18,29]. The results presented by these authors are consistent with those obtained in the present study. Similar observations have been reported by Fowcett et al., who reviewed studies on the preoperative administration of preparations containing polycarbohydrate [28,30]. The positive perception of this therapeutic approach results from the reduction in negative emotions that typically accompany hospitalization and are often intensified—such as psychomotor agitation or crying in children awaiting surgery [1,27,31]. The presented research results should be interpreted with caution due to the heterogeneity of the study group orthopedic patients with various indications for surgery.

It should be emphasized that few studies have been identified in the literature on the administration of polycarbohydrate preparations to orthopedic patients [32,33,34]. Preliminary reports on tolerance and metabolic response in thoracic surgery and pediatric urology were published by Gawecka et al., whose observations are consistent with the findings of the present study. Metabolic preparation is not routinely used in the pediatric patient population in Poland, and research in this area remains limited [26]. The presented study was based on literature concerning pediatric patients from Europe, North America, Australia, and Asia [35,36,37]. The issue of preoperative fasting and oral polycarbohydrate intake is becoming increasingly important in the context of the ERAS protocol and the role of the interdisciplinary team—particularly the role of the nurse in implementing the principles of preoperative patient preparation [37,38,39]. It should also be noted that the metabolic response of children to stress, treatment, or injury may differ significantly from that of adults [33]. An additional aspect considered in relation to the administration of polycarbohydrate preparations before surgery is the potential impact on the workload of medical personnel. Research conducted by the Australian Nurses Association, which evaluated the nursing workload required to fulfil ERAS protocol recommendations, including the administration of oral polycarbohydrate solutions, clearly indicates that proper implementation does not increase nursing work time but significantly improves the quality of care and patient satisfaction [40]. This highlights an important area for interventions aimed at improving the comfort of pediatric surgical patients in hospitals and requires further research on this issue.

## 5. Limitations

Although all patients were given general anesthesia, the use of different medications during anesthesia could be a source of discrepancies.

The study group was also not homogeneous in terms of indications for surgical intervention. The study included patients who qualified for orthopedic surgery after an injury, people with congenital defects and post-traumatic deformities of the limbs, and patients undergoing surgery to remove material used to stabilize a fracture, so the time of surgery varied. Additional differences concerned the method of admission—children admitted in emergency and those admitted as part of planned procedures. None of the patients was in a life-threatening condition, so no one was given anesthesia without a 6 h fast preceding it.

Another limitation is the single glucose level test, conducted only before anesthesia, which is due to the pilot nature of the study.

## 6. Conclusions

1. Glucose levels were higher in patients who received a polycarbohydrate preparation compared with those who did not.
2. The administration of polycarbohydrate preparations influenced the feelings of thirst and hunger in the postoperative period among children and adolescents.
3. Oral administration of a polycarbohydrate preparation up to 2 h before anesthesia did not cause symptoms of regurgitation in children and adolescents.
4. Patients who received an oral polycarbohydrate preparation tolerated the waiting period before anesthesia better.
5. Preoperative fasting is, apart from venous access placement, the most stressful aspect of hospitalization for children and adolescents.
6. The supply of oral polycarbohydrate formulations may have a positive effect on pediatric patients in the perioperative period.

## Figures and Tables

**Table 1:** Comparison of pre-treatment glucose levels in control and study group of children.

Level	Control Group			Study Group			Test *t*		
	*N*	*M*	*SD*	*N*	*M*	*SD*	*t*	*df*	*p*
Venous blood glucose	50	73.46	7.48	50	83.22	9.90	−5.56	98	<0.001

*N*—number of observations; *M*—mean; *SD*—standard deviation; *t*—Student’s *t*-test value; *df*—degrees of freedom; *p*—level of statistical significance.

## Data Availability

The data are not publicly available due to ethical restrictions related to the protection of sensitive personal and health-related information. Access to the dataset may be granted by the corresponding author upon reasonable request, in accordance with ethical approval.
